# Incorporating social determinants of health into individual care—a multidisciplinary perspective of health professionals who work with people who have type 2 diabetes

**DOI:** 10.1371/journal.pone.0271980

**Published:** 2022-08-08

**Authors:** Amanda Frier, Sue Devine, Fiona Barnett, Kris McBain-Rigg, Trisha Dunning

**Affiliations:** 1 College of Public Health, Medical and Veterinary Sciences, James Cook University, Townsville, Queensland, Australia; 2 College of Healthcare Sciences, James Cook University, Townsville, Queensland, Australia; 3 Centre for Quality and Patient Safety Research, Faculty of Health, Deakin University and Barwon Health Partnership, Melbourne, Victoria, Australia; Vanderbilt University Medical Center, UNITED STATES

## Abstract

Social determinants of health (SDoH) and type 2 diabetes mellitus (T2DM) are interrelated. The prevalence of T2DM is increased amongst those with suboptimal SDoH. Poor SDoH can also negatively impact T2DM self-management. Social determinants of health are mostly considered at population and community levels, rather than individually or clinically. This qualitative study combines the perspectives of a multidisciplinary cohort of health professionals to identify and explore the impact of social determinants on self-management, and ways they could be incorporated into individual clinical care. Purposively selected participants chose to partake in an in-depth, semi-structured, one-on-one interview or focus group. Data were analysed, and themes identified using a combination of deductive and inductive thematic analysis. Fifty-one health professionals volunteered for the study. Two small focus groups (n = 3 and n = 4) and 44 one-on-one interviews were conducted. The identified themes were: 1) Support for incorporating SDoH into T2DM care, 2) Effect of SDoH on T2DM self-management, 3) Identifying and addressing social need, 4) Requirements for incorporating SDoH into T2DM individual clinical care. Health professionals reported that poor social determinants negatively affect an individual’s ability to self manage their T2DM. Person-centred care could be enhanced, and people with T2DM may be more likely to achieve self-management goals if SDoH were included in individual clinical care. To achieve successful and sustained self-management for people with T2DM, health professionals require a thorough understanding of T2DM and the effect of social determinants, respect for client privacy, client trust and rapport, effective communication skills, validated tools for assessing SDoH, team champions, teamwork, ongoing education and training, adequate resources, guiding policies and procedures, and management support. Incorporating SDoH into individual, clinical care for people with T2DM was strongly supported by health professionals. If embraced, this addition to care for individuals with T2DM could improve self-management capacity and enhance person-centred care.

## Introduction

Health professionals (HPs) strive to provide quality, person-centred care for people with diabetes [1]. Person-centred care is especially beneficial when working with people who live in disadvantaged situations. Socially disadvantaged people have a higher prevalence of type 2 diabetes mellitus (T2DM), and face more barriers when self managing their diabetes [2–4]. Social disadvantage is an accumulation of suboptimal social determinants of health (SDoH) which include; income, employment, housing, education and health literacy, transport, social support and access to healthcare [2, 5].

Social determinants of health are typically addressed at a population level and aim to achieve sustained health equity, social justice and generational improvement of people’s lives [2, 6, 7]. Momentum towards this was strengthened following the 2008 ‘Commission on Social Determinants of Health (CSDH)’ [7]. The commission’s overarching principles guide action on SDoH at global, national and local levels. Upstream, population approaches directed at political, societal and economic improvement are the foundation of the CSDH’s purpose of lasting health equity and social justice [2, 7, 8].

Advocacy and action towards health equity and social justice is progressing, however until permanent improvement of people’s lives is achieved, a substantial proportion of the population will continue to live in socially disadvantaged situations, with poor SDoH. As a consequence, their ability to manage chronic conditions, such as T2DM is impeded.

Similar to the clear evidence base supporting action on SDoH [2, 6, 7], the association between SDoH and T2DM is well-established [4, 9–11]. While assessing and addressing nonclinical issues such as SDoH is not the major focus in clinical T2DM settings, the necessity to do so is emphasised by the entwinement of socio-economic factors and overall health [2, 7, 12]. To date, clear guidance on how to include SDoH into individual, clinical healthcare is scarce. This may stem from a shortfall in supportive policies and organisational guidance for taking action on SDoH in healthcare settings [13]. However, international momentum towards screening, and addressing SDoH issues on an individual level in clinical healthcare settings has begun [14–20]. These approaches could provide helpful guidance for the incorporation of SDoH into the clinical management of T2DM.

Once contextualised to specific regions, diabetes services could embed SDoH as part of usual clinical care for individuals with T2DM. The integration of SDoH into T2DM clinical practice could provide health professionals with insight into their client’s life circumstances, and broader SDoH related barriers to T2DM self-management. Thoroughly understanding an individual’s SDoH, and their impact on self-management, may assist health professionals to deliver a more comprehensive person-centred intervention [1] and lead to greater improvement in health outcomes for their clients.

The current study combined viewpoints from the multidisciplinary range of health professions involved in diabetes care. The aims were to investigate how SDoH affects T2DM self-management, and ways to incorporate SDoH issues into clinical, individual care. The findings will contribute to the body of evidence supporting the affliction of suboptimal SDoH on T2DM [21]. Furthermore, they will inaugurate momentum towards incorporating SDoH into the usual clinical care of people with T2DM.

## Methods

This qualitative study used an exploratory, descriptive approach [22]. The intent was to increase understanding of how SDoH affects the self-management ability of people with T2DM, and the relevance for HP’s who work with them. The qualitative design also enabled HP experience and insight to inform how SDoH could be incorporated into usual clinical care for people with T2DM.

### Research aims

The aims of the study were to draw on the perspectives of health professionals who work with people with T2DM to:

1. Identify and explore SDoH related issues, and their repercussions on people with T2DM and the health professionals that work with them.
2. Identify and explore how to include SDoH into usual clinical care for people who have T2DM.

### Situating the researcher

The primary researcher (AF) is a dietitian and diabetes educator. She has provided diabetes care throughout rural, remote and regional North Queensland, Australia (NQ) for over 19 years. This experience has provided exposure to the SDoH challenges people with T2DM can endure, and has motivated her to investigate the topic reported in this paper. A reflexive mindset was adopted to ensure self-reflection and self-awareness of personal context and experiences were considered during all stages of the research [23].

### Setting

Health professionals who work with people who have T2DM, from five health services in NQ participated in the study. Three were government services, one was a not-for-profit organisation and one a fee-for-service provider. These health services provided both community centre-based and outreach diabetes care to rural, remote and regional communities across the NQ region.

### Participant recruitment

Employees from the five NQ diabetes service providers were purposively selected. Purposive sampling assured a sample reflecting the wide range of HP’s involved in diabetes care were recruited [22]. AF commenced recruitment by conducting face-to-face, introductory meetings with managers at each diabetes service. A detailed explanation of the research project and information sheets were provided. Management briefed their staff about the study, provided information sheets and invited voluntary participation. Upon volunteering, suitable dates and times for study involvement were negotiated between AF, the staff member and their manager. To achieve sample heterogeneity AF maintained regular communication with managers via phone, email and face-to-face meetings to encourage recruitment activities until all disciplines involved in T2DM care were represented.

### Data collection

Participants chose to take part in either an in-depth, semi-structured, one-on-one interview or focus group. AF conducted all interviews and focus groups to maximise consistency between the two data collection methods. An informal, conversational style of interviewing was used for both one-on-one interviews and focus groups. Conducting the interviews and focus groups in meeting rooms, rather than clinic settings, added to a relaxed and comfortable environment for study participants. Demographic information, profession and years of experience were captured using a written questionnaire prior to each interview/focus group. AF and the second researcher (SD) developed the interview guide which was informed by a literature review on the topic [24]. A third researcher (FB) reviewed the interview guide for participant suitability and research question adherence, with no major changes suggested. Open-ended questioning elicited understanding of SDoH in relation to T2DM, and insights on how SDoH could be incorporated into clinical care. The interview/focus group questions were piloted with two credentialled diabetes educators. Data saturation was inferred when no new responses from HPs emerged. Interviews and focus groups were audio-recorded, and informed consent was gained immediately before commencement of each interview/focus group.

### Data analysis

Data analysis was guided by the Braun and Clarke’s six steps for thematic analysis [25, 26]. All audio-recordings were transcribed and then reviewed manually by AF. A sample of these transcripts were reviewed independently by SD, enabling independent theme identification, and increased study rigour. Transcripts were thematically analysed using QSR NVivo v12 (QSR International; http://www.qsrinternational.com/nvivo) for data management. Deductive and inductive data analyses were conducted simultaneously [25, 27]. The deductive analysis was based on a framework of well-known SDoH (Table 1) [2]. A phenomenological approach to the inductive analyses facilitated deep exploration of health professional experiences [22, 26] of working with people who have T2DM, through a SDoH lens, and how these insights could be incorporated into clinical care. Analytical discussions on coding and theme development were conducted between AF, SD and the fourth researcher (KMR). Member checking involved providing a detailed explanation of the identified themes to the diabetes service managers via email. Managers then communicated the themes to study participants, and invited feedback. In addition, two feedback sessions were provided to study participants (n = 8 and n = 6). There were no recommended changes to the identified themes.

**Table 1:** SDoH framework used for deductive analysis.

Social Determinants of Health
Addiction
Early life
Economic status (income)
Education
Employment
Food security
Healthcare access
Housing
Social exclusion
Social support
Stress
The social gradient
Transport

### Ethics approval

Ethics approval was granted by the Human Research Ethics Committee of Queensland Health (HREC/18/QTHS/128) and James Cook University, Australia (H7480).

## Results

### Participant characteristics

Forty-four one-on-one interviews and two small focus groups (n = 3 dietitians) and (n = 4—one podiatrist and three registered nurse/credentialled diabetes educators) were conducted (total 51 participants). Participants included ten males and 41 females aged between 22–64 years (median 38.5 [IQR 29–50]). Years of diabetes experience ranged from 0.25–40 years (median 8 [IQR 4–15]). Participating disciplines included endocrinologists, general practitioners, a medical registrar, a public health physician, an enrolled nurse, a practice manager, diabetes educators, dietitians, podiatrists, clinical exercise physiologists, physiotherapists, psychologists, social workers, occupational therapists, a speech pathologist, nurse practitioners, and nurse navigators. Participant qualifications included certificate level, diploma, undergraduate and postgraduate, with most participants holding postgraduate qualifications. Table 2 displays participant discipline, role and demographic information. All study participants were non-Indigenous as the perspectives of Australian Aboriginal and Torres Strait Islander health workers are published in a separate paper [28].

**Table 2:** Health professional demographic and career information.

Factor	Number *(N = 51)*
**Demographic factors**	
Female	41 (80%)
Male	10 (20%)
Median years of age (IQR)	38.5 (29–50)
**Level of education**	
Postgraduate	34 (67%)
Undergraduate	15 (29%)
Diploma of Nursing	1 (2%)
Cert IV (Business)	1 (2%)
**Years of experience and disciplines included**	
Median years of diabetes experience (IQR)	8 (4–15)
Registered Nurse/CDE*	9 (17.6%)
Dietitian	7 (13.7%)
Podiatrist	4 (7.8%)
Endocrinologist	3 (5.9%)
General Practitioner (clinic specialising in diabetes)	3 (5.9%)
Physiotherapist	3 (5.9%)
Occupational Therapist	3 (5.9%)
Registered Nurse / Nurse Navigator	2 (3.9%)
Registered Nurse / Diabetes Educator (not credentialled)	2 (3.9%)
Clinical Exercise Physiologist	2 (3.9%)
Psychologist	2 (3.9%)
Social Worker	2 (3.9%)
Public Health Physician (specialist diabetes centre)	1 (2%)
Medical Registrar (specialist diabetes & endocrinology centre)	1 (2%)
Nurse Practitioner / CDE	1 (2%)
Nurse Practitioner / Nurse Navigator / CDE	1 (2%)
Clinical Nurse Consultant / Registered Nurse / CDE	1 (2%)
Endorsed Enrolled Nurse (specialist diabetes centre)	1 (2%)
Dietitian / CDE	1 (2%)
Speech Pathologist	1 (2%)
Practice Coordinator (clinic specialising in diabetes)	1 (2%)

* Credentialed Diabetes Educator

### Focus group and interview findings

During data analysis, the issues discussed during the focus groups and the one-on-one interviews were similar. In addition, the number and size of the focus groups (n = 3 and n = 4) were very small. Accordingly, data from the focus groups and interviews were amalgamated and themes were identified collectively.

The identified themes were;

1. Support for incorporating SDoH into T2DM care
2. Effect of SDoH on T2DM self-management
3. Identifying and addressing social need
4. Requirements for incorporating SDoH into T2DM individual clinical care

#### Support for incorporating SDoH into T2DM care

Overwhelmingly HPs indicated that understanding, and incorporating SDoH into clinical care for people with T2DM would be beneficial to practice, and is ultimately necessary for comprehensive T2DM care. Study participants felt that increased awareness of SDoH related self-management barriers would lead to more individualised, achievable and effective management strategies, based on each person’s social circumstances.

**HP 2** (Endocrinologist)

So those are the sort of things [SDoH related barriers], that if we know, what are the barriers. It makes it easier for us to go ahead and design a treatment that suits the person….. and I think, we’ve got enough in our hands at the moment, the various medications that we have, we can individualise a treatment, and design a treatment that meets their needs.

Furthermore, many HPs reported a deflated professional self-worth because they were unable to help people with T2DM achieve their self management goals, because of SDoH related barriers.

**HP 19** (Registered Nurse / Credentialled Diabetes Educator)

Sometimes you feel a bit despondent, with, you know. Not with the work you do with people, I always love to work with people, and feel like you can possibly help them in a positive way, but sometimes I just feel that I can’t really make a big difference, because there’s these things…[SDoH related barriers].

Whilst the value of incorporating SDoH into the clinical care of T2DM was clear, HPs expressed privacy related concerns that may arise when exploring the personal, social issues of people with T2DM. Table 3 provides a summary of the privacy issues raised by HPs.

**Table 3:** Privacy related issues raised by HP’s.

HP concerns for the privacy of people with T2DM
Invasion of privacy by delving into personal issues
Raising personal and sensitive issues—“hitting nerves”
People with T2DM may choose not to divulge SDoH information due to embarrassment and pride
Inability for people with T2DM to see the correlation between their SDoH and T2DM self-management
Potential for people with T2DM to become defensive when raising personal issues
People with T2DM may feel HPs are being nosy

#### Effect of SDoH on T2DM self-management

T2DM self-management was unquestionably affected by people’s SDoH. HPs described two layers of effect. The first was on the personal ability of people with T2DM to achieve self-management goals. The deductive analysis framework [2] helped to identify that deficits in any SDoH can create barriers for T2DM self-management. However, the most-reported hindrances on self-management were; limited access to suitable transport, financial concerns and minimal social support. As a result, people with T2DM could not attend diabetes related appointments, afford healthy food and medications, or rely on support people to assist with self-management difficulties. Most notably, HPs felt it was the aggregation of poor SDoH that leads to suboptimal self-management, rather than the result of one single social determinant.

**HP 21** (Registered Nurse / Credentialled Diabetes Educator)

I mean it’s, overcrowding, people haven’t got employment, people haven’t got access to food… throw in the gambling, throw in the drugs… and oh my God it’s a nightmare… It’s just an absolute nightmare!

**Fig 1:**
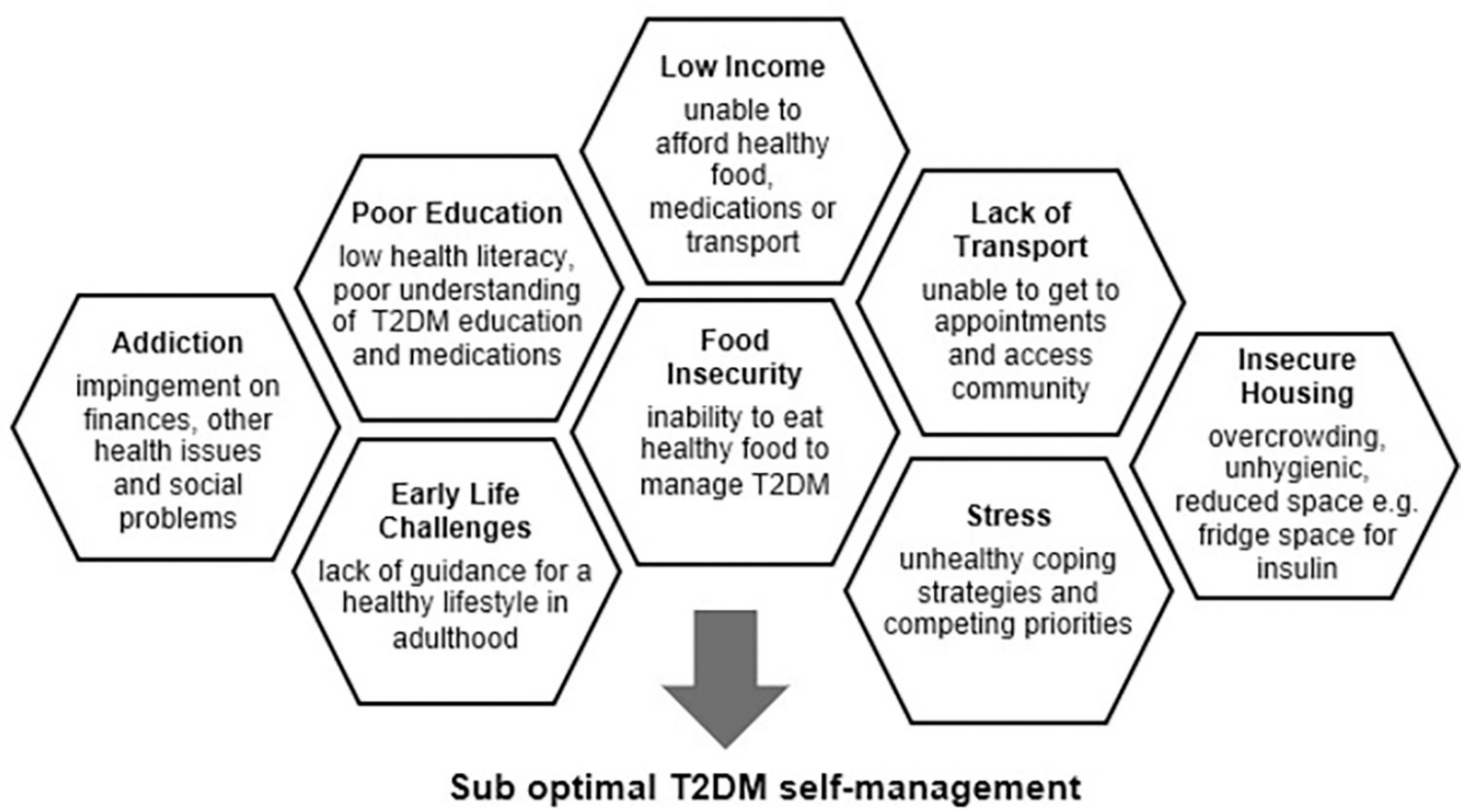
Combination of SDoH issues contributing to sub-optimal T2DM self-management.

Fig 1 displays examples of the interconnected SDoH circumstances encountered in practice by HPs. The HPs described these individual SDoH issues as combining, and collectively contributing to suboptimal T2DM self-management.

The second effect was not a direct consequence of an individual’s SDoH, instead, it was related to health service processes, and how these can exacerbate negative SDoH, specifically healthcare access. The role health services can play in hindering healthcare access was identified in this study. HPs frequently raised high ‘failure to attend’ (FTA) rates. While a small number of HPs felt it was purely people with T2DM choice to attend their appointments (or not), most indicated health services had a responsibility to facilitate access to healthcare to reduce high FTA rates.

**HP 12** (Registered Nurse / Nurse Navigator)

I think the other thing is those patients would be labelled as FTA’s, and they may have called up and spoken to someone, but because there was limited, I suppose, ability to put–[support], around that, they’d be marked as FTA. So then they’d sort of get labelled as a patient with FTA’s all the time, but that’s actually not the issue, it’s that they have actually genuinely tried, or they wouldn’t see the letter in time.

#### Identifying and addressing social need

All HPs agreed there were two main aspects to incorporating SDoH into T2DM clinical care. These were; identifying the social need of people with T2DM, and addressing this identified social need.

#### Identifying social need

Participants suggested SDoH could be assessed using a validated tool. Home visits to witness living environments were also discussed; however many HPs aired concerns that home visits would add time and resources to an already excessive workload. They felt, something quick and easy was required to assure SDoH assessments were part of usual practice such as a checklist that identified the issues relevant to each person with T2DM.

Participant consensus confirmed the initial SDoH assessment should be conducted when the people with T2DM first engages a T2DM service. It was felt that this information would inform all areas of T2DM care.

**HP 9** (Nurse Practitioner / Credentialled Diabetes Educator)

So, and on an initial assessment [first T2DM appointment] it’s a valid thing to do, and it sets the tone for conversation to, around the client. You know, a more personalised approach to that person.

Timeframes for repeated SDoH assessment varied from three months to two years. Some HPs commented on the many life events that can occur for people with T2DM, and suggested this be the indicator for reassessment.

**HP 8** (Medical Registrar)

If any big issues comes up then it probably needs to be reassessed [SDoH reassessment], like if they have a marriage breakdown or something.

When determining who should identify social need, HPs described the necessity of rapport, trust, effective communication, and an understanding of T2DM. They concurred that a qualified HP should be responsible for conducting the SDoH assessment, rather than administration or a community worker. Expanding this concept, HPs with more experience or higher positions within the T2DM team attested that it was each and every HPs’ responsibility because SDOH is integral to person-centred T2DM care.

**HP 14** (Registered Nurse / Credentialled Diabetes Educator)

At the end of the day. It’s what we should all be doing, but you know from that person centred approach, all of the social determinants of health come into that, don’t they!

#### Addressing identified social need

HPs expressed that assurance of improved T2DM outcomes would increase the likelihood that incorporating SDoH into clinical care would be embraced. They also felt understanding SDoH related self-management barriers would only be useful if corrective action could be taken on the identified issue.

**HP 3** (Endocrinologist)

I think if a patient [person with T2DM] has already had a social assessment. It would only be of benefit if something is done about it.

Substantiating this, was unanimity in having a position within the T2DM team, solely responsible for assisting people with T2DM to navigate identified SDoH related barriers. The stipulation of being a team member was to assure regular feedback and continuity of care. For similar reasons, HPs also stressed a need for external referral feedback, and lamented its current shortfall.

As with identifying SDoH related barriers; trust, rapport, communication and T2DM understanding were rudimentary to addressing social need. In addition, experience in the social arena and strong community networks and relationships were paramount. As such, the two most prominently suggested HPs were nurse navigators and social workers.

**HP 1** (Public Health Physician)

The other thing that was a light in the darkness, was nurse navigators. The principle of that whole thing is identifying the individuals needs in their life, not their medical need. So a lot of work that happens there, by the navigators [nurse navigators], is around their social, psychological, situation, to get that sorted out, and then they can address their healthcare.

**HP 43** (Psychologist)

I think social work is in a good place to answer, I guess, a lot of those kinds of practical questions that lie outside of my scope of knowledge and the kind of connections I have with the community. Social work has a lot of those things…Yeah I think social work’s important. I think social work, particularly in the practical side of things, of helping people to access services, and access things, can be, is very valuable I think.

When the nurse navigators and social workers in the study described their roles and responsibilities, congruity with the stated requirements was evident, and therefore confirmed their suitability to the role. Furthermore, they agreed with this proposition.

#### Requirements for incorporating SDoH into T2DM individual clinical care

For SDoH to be sustainably incorporated into clinical care for people with T2DM, HPs felt there were some fundamental requisites. Management support; team champions, guiding policies and procedures; ongoing education and training; teamwork, and adequate resources (staffing, time, intervention tools, and referral follow-up processes) were all described as facilitators to incorporating SDoH into the clinical care of people with T2DM.

## Discussion

Suboptimal SDoH and T2DM are often mutually inclusive [29]; however, they are usually addressed separately, with disparate approaches for mitigating their undesirable health effects. SDoH are usually considered at a population level, and T2DM at an individual, clinical level [2, 3]. This study sought to investigate HP perspectives on how SDoH could be incorporated into the clinical care of individuals with T2DM, and to understand the affliction of SDoH on T2DM. The diverse range of HPs in this study is representative of the multidisciplinary approach to T2DM care [3] and therefore adds to the study’s credibility.

The findings reflected an interconnection between the coalescence of poor SDoH, and suboptimal T2DM self-management. HPs in the current study expressed a strong desire to include SDoH into clinical care for people with T2DM. This appeal was bolstered in a recent pilot study by Neadley et al. [30]. The pilot study was conducted in a disadvantaged population of Adelaide, Australia. Study participants (patients) felt collecting SDoH information at an individual level would benefit both patients and healthcare providers by improving communication, identifying patient service needs, and highlighting previously unrecognised issues. Furthermore, the authors reported that the patients were comfortable divulging sensitive information [30]. This patient assurance may appease the people with T2DM privacy concerns expressed by HPs in the current study.

Concurrence between patients in the study by Neadley et al. [30] and HPs in the current study was also noted in their equivalent concern for healthcare provider time constraints. Participants in both studies inferred a person dedicated to collecting SDoH related information was a possible redress. Patients suggested a researcher [30], and HPs felt a team member with experience in T2DM was required. Though differing in who should collect the information, the corresponding perspective of a dedicated role to assist with time management emphasises the imperative of considering HP time and workload when incorporating SDoH into clinical care. Furthermore, a dedicated team member may indicate management commitment and support, and increase the likelihood of appropriate resources and tools to enhance this approach.

Health professionals in the current study felt that any tool used to collect individual SDoH information should be validated. The social health screening (SHS) tool used by Neadley et al. was developed with input from patients and clinicians, who confirmed it’s appropriateness for use. Though the tool has been tested in a “proof-of-concept study”, it has not been validated, and the authors recommend further pilot studies to discern efficacy [19, 30]. The SDoH related issues it is designed to identify are the same as those experienced by people with T2DM [3, 4], so conceivably, it would also apply to this cohort. However, if the SHS tool were applied in a T2DM specific, community-clinical setting, it would require validation.

The consistency between patients and HPs asserts merit for collecting SDoH related information at an individual and clinical level. Though the study by Neadley et al. [30] did not exclusively include people with T2DM, and was in an inpatient setting, transferability to all chronic diseases and community-clinic settings is easy to conceive. This notion was corroborated by Kusnoor et al. [31] whose study findings supported the feasibility of collecting SDoH and behavioural information from individuals in community-clinical settings.

The value of collecting SDoH information from people with T2DM could be enhanced if the self-management barriers they incite became surmountable. In addition to a team member being dedicated to collecting SDoH information, HPs in the current study felt the person in this role could also assist people with T2DM in taking action on the identified SDoH issues. Andermann [32] also advocated this and suggested patient facilitators or navigators could assist with support service access. The HPs suggestion of nurse navigators or social workers suitability to this role is indeed logical; however, it would depend on the model of care the health service is operating within. For example, if nurse navigators or social workers did not work within the team, an alternate team member would be required. Subsequently, all team members should be trained to ask about SDoH, and assure the identified issues are actioned, either by referral, connection with appropriate services, or other means such as direct support for the identified SDoH issue [19, 32]. This sentiment also emerged in the current study and reiterates the importance of a whole of team approach that includes a dedicated role to focus on an individual’s SDoH circumstances.

If people with T2DM were able to achieve their self-management goals, the feelings of professional ineffectiveness expressed by HPs in the current study could be ameliorated. The futility and despondence they reported may relate to an inability to respond to SDoH barriers, as was identified in a study conducted among Australian primary health care services. The authors suggest this may be due to the discrepancies between comprehensive and selective primary health care [13]. Selective primary health care focuses on individuals, behaviour and disease, whereas comprehensive primary healthcare involves a broader view of health and considers the underlying disease causes, i.e. SDoH [33]. The predicament of low professional self-worth reported by HP’s in the current study may also be underpinned by the conflict between selective and comprehensive primary healthcare. The services they work within are oriented towards a selective approach; however, addressing SDoH issues requires a more comprehensive course of action [33].

T2DM management is usually centred around biomedical and behavioural interventions [3] which is converse to the comprehensive primary health care approach required for SDoH resolve [34]. Extending the biomedical view of T2DM care by incorporating SDoH into the individual, clinical management for people with T2DM may lean towards selective primary health care; however, acknowledging and acting on the influence of SDoH, in clinical settings, is an additional step towards addressing the impedance adverse social circumstances can have on optimal health. Importantly though, it should not the intended to replace a comprehensive approach to primary health care.

The focus of this study was on the SDoH of individuals in clinical settings, and how SDoH influence the self-management of T2DM. However, there was also the incidental emergence of health provider processes compounding SDoH related barriers for people with T2DM. In this case healthcare access was allegedly inhibited because of ineffective appointment making processes that resulted in high FTA rates. Access to healthcare is a well-known SDoH [4] and is often accounted for in health equity initiatives [35–37]. The majority of effort towards improving healthcare access pivots around reducing barriers such as lack of transport, affordability and distance from healthcare with little, if any, focus on the barriers health services themselves create [37]. Therefore, if incorporating the SDoH of individuals with T2DM into clinical care is to be optimised, additional consideration of institutional barriers is fundamental.

## Limitations

This study was conducted in rural, remote and regional communities throughout NQ, Australia, and therefore may not be representative of other T2DM services within Australia or internationally. In addition, participants in the current study worked under varying health service delivery models, i.e. centre-based and outreach services. Though all HPs conveyed comparable views about the influence of SDoH on T2DM self-management, and how to incorporate them into individual care, this perspective may not necessarily be true for every health service delivery model. If contextualising an approach that includes SDoH into the clinical care of individuals with T2DM, the model of health care delivery and the region in which it is applied would need to be considered. Lastly, the viewpoints of pharmacists (who also work with people who have T2DM) was not captured due to their employment being outside the boundaries of ethics requirements. Given the overall consensus of the relatively large multidisciplinary sample in this study, it is likely that pharmacists’ perspectives would be consistent with the reported findings.

## Conclusion

A multidisciplinary cohort of health professionals who work with people who have T2DM in NQ, Australia provided insight into how SDoH could be incorporated into individual, clinical care for people with T2DM. There was strong acknowledgment of the influence SDoH can have on T2DM self-management, and ensuing support for this novel approach to T2DM care. The study revealed that successful and sustainable implementation requires management support and a team member dedicated to this role. In addition, formal incorporation of SDoH into the clinical care of individuals with T2DM would require validated tools that supported the collection of appropriate information, and subsequent action on the identified SDoH related barriers to T2DM self-management. The findings of this study suggest that collecting SDoH information, and appropriately intervening to assist people with T2DM to surmount the barriers they impose would contribute to the person-centred care required for optimal T2DM self-management [1].

## Supporting information

S1 File Interview/Focus group guide. (DOCX)

S2 File Demographic and career questionnaire. (DOCX)
