# Supplementary material for: Incorporating social determinants of health into individual care—a multidisciplinary perspective of health professionals who work with people who have type 2 diabetes
Source: PLoS One. 2022 Aug 8;17(8):e0271980. doi: 10.1371/journal.pone.0271980 (PMC9359576; doi:10.1371/journal.pone.0271980)
Supplement: S1 File — (DOCX) [file pone.0271980.s001.docx]

**Interview / Focus Groups Question Guide**

***Health professionals working with people who have T2DM***

1. Introductions and rapport building (aim to create a relaxed environment) e.g. how long have you worked in diabetes, experiences etc
2. Brief explanation of the focus group purpose
3. Can you please talk about some of the nonclinical barriers that people tell you about (or that you are aware of) with regard to managing T2DM?
   - *Depending on the responses, probe and facilitate discussion around the following.*

| **Commonly known social determinants of health** (not necessary to use SDoH specific terms) | | | | | |
| --- | --- | --- | --- | --- | --- |
| Addiction | Economic Status (Income) | Employment | Housing and home situation | Health Literacy (education) | Healthcare Access |
| Social Exclusion | Social Support e.g. childcare, supportive family and friends or other social groups | Stress | Transport | Food Security | Early Life  (prevention of T2DM in future generations) |

1. Do you feel that these (as discussed above) could be considered, and then addressed to help people manage their T2DM?

*- If yes ask questions 5*

*- If no explore*

1. How could these (as discussed above) be considered and addressed in T2DM management?
   - *While asking these questions probe and facilitate discussion by asking*

| Who | When |
| --- | --- |
| What | How |
| Where | Why |

- *If necessary probe and facilitate discussion around both assessing SDoH related issues and approaches for addressing them*

**5a.** If the final consensus is ‘yes’ these factors should be considered and addressed in the management of T2DM.

- - What barriers might there be ……?
  - What facilitators could there be…..?

**5b.** If the final consensus is ‘no’ these factors should not be considered and addressed in the

management of T2DM…. explore this further.

1. Which groups in the community seem to be the most disadvantaged because of the issues discussed above?
   - *Facilitate discussion on …..who… what....why…why not…in what way?*

**7** . Finalise discussion and ask if participants they have any questions or comments at all.

**8** Inform participants will happen next in the research project….

THANK THEM VERY MUCH !
