# Supplementary material for: Incorporating social determinants of health into individual care—a multidisciplinary perspective of health professionals who work with people who have type 2 diabetes
Source: PLoS One. 2022 Aug 8;17(8):e0271980. doi: 10.1371/journal.pone.0271980 (PMC9359576; doi:10.1371/journal.pone.0271980)
Supplement: S2 File — (DOCX) [file pone.0271980.s002.docx]

**W*ritten Questions for Health Professionals***

1. What is your profession?

------------------------------------------------------------------------------------------------------------

1. Are you a credentialled diabetes educator?

------------------------------------------------------------------------------------------------------------

1. Are you male or female ?

------------------------------------------------------------------------------------------------------------

1. What is your age ?

------------------------------------------------------------------------------------------------------------

1. What is your level of education?

**certificate - diploma - degree - postgraduate studies**

1. How long have you worked with people who have diabetes?

------------------------------------------------------------------------------------------------------------

1. Do you work in a ‘diabetes centre’ based facility? i.e. hospital based service or community based service.

------------------------------------------------------------------------------------------------------------

1. Do you provide outreach diabetes services? --------------------------------------------------
   - Face-to-face
   - Telehealth
